# Housekeeping Gene Stability in Adipose Mesenchymal Stromal Cells Cultivated in Serum/Xeno-Free Media for Osteoarthritis

**DOI:** 10.3390/cells13020167

**Published:** 2024-01-16

**Authors:** Enrico Ragni, Simona Piccolo, Paola De Luca, Michela Taiana, Giulio Grieco, Laura de Girolamo

**Affiliations:** Laboratorio di Biotecnologie Applicate all’Ortopedia, IRCCS Istituto Ortopedico Galeazzi, Via Cristina Belgioioso 173, 20157 Milano, Italy; enrico.ragni@grupposandonato.it (E.R.); simona.piccolo@grupposandonato.it (S.P.); michelamaria.taiana@grupposandonato.it (M.T.); giulio.grieco@grupposandonato.it (G.G.); laura.degirolamo@grupposandonato.it (L.d.G.)

**Keywords:** housekeeping genes, mesenchymal stromal cells, osteoarthritis, regenerative medicine, fetal bovine serum, xeno-free/serum-free media

## Abstract

Among the available therapeutics for the conservative treatment of osteoarthritis (OA), mesenchymal stromal cells (MSCs)-based products appear to be the most promising. Alongside minimally manipulated cell-based orthobiologics, where MSCs are the engine of the bioactive properties, cell expansion under good manufacturing practice (GMP) settings is actively studied to obtain clinical-grade pure populations able to concentrate the biological activity. One of the main characteristics of GMP protocols is the use of clinical-grade reagents, including the recently released serum-free/xeno-free (SFM/XFM) synthetic media, which differ significantly from the traditional reagents like those based on fetal bovine serum (FBS). As SFM/XFM are still poorly characterized, a main lack is the notion of reliable housekeeping genes (HKGs) for molecular studies, either standalone or in combination with standard conditions. Indeed, the aim of this work was to test the stability of five commonly used HKGs (*ACTB*, *EF1A*, *GAPDH*, *RPLP0*, and *TBP*) in adipose-derived MSCs (ASCs) cultivated in two commercially available SFM/XFM and to compare outcomes with those obtained in FBS. Four different applets widely recognized by the scientific community (NormFinder, geNorm, comparative ΔCt method, and BestKeeper) were used and data were merged to obtain a final stability order. The analysis showed that cells cultured in both synthetic media had a similar ranking for HKGs stability (*GAPDH* being best), albeit divergent from FBS expanded products (*EF1A* at top). Moreover, it was possible to identify specific HKGs for side by side studies, with *EF1A*/*TBP* being the most reliable normalizers for single SFM/XFM vs. FBS cultured cells and *TBP* the best one for a comprehensive analysis of all samples. In addition, stability of HKGs was donor-dependent. The normalization effect on selected genes coding for factors known to be involved in OA pathology, and whose amount should be carefully considered for the selection of the most appropriate MSC-based treatment, showed how HKGs choice might affect the perceived amount for the different media or donor. Overall, this work confirms the impact of SFM/XFM conditions on HKGs stability performance, which resulted similarly for both synthetic media analyzed in the study.

## 1. Introduction

Osteoarthritis (OA) is the most common degenerative pathology of the joints, affecting 250 million people worldwide [1]. Ageing of the population and obesity are actually the major drivers of new cases of OA, with an expected increased impact on health services and societies. Together with the classical definition of OA as a disease centered on the changes in the articular cartilage, nowadays it is considered a pathology of the whole joint, encompassing subchondral bone, ligaments, capsule, and synovial membrane [2]. For these reasons, OA treatment is a complex strategy. The initial management is conservative, with pharmacological (nonsteroidal anti-inflammatory drugs (NSAIDs) or opioids) and infiltrative (hyaluronic acid (HA)) approaches. When noninvasive therapy fails, the surgical strategy and prosthetic interventions are the last option [3]. To delay or possibly halt the need of prosthesis, in the last years a new category of infiltrative approaches has emerged, based on the use of orthobiologics [4], including platelet-rich plasma (PRP), adipose-tissue-derived products such as stromal vascular fraction (SVF) or microfragmented adipose tissue (MFAT), and bone marrow aspirate concentrate (BMAC). SVF, MFAT, and BMAC base their biological activity on the resident population of mesenchymal stromal cells (MSCs), able to release soluble factors and extracellular vesicles (EVs) to modulate inflammatory populations [5], such as macrophages, and stimulate tissue resident progenitor cells [6]. Due to the ease of harvest and the higher number of MSCs compared to bone marrow, adipose-derived products are a privileged option [7].

Recently, to increase therapeutic power, the idea of injecting expanded MSCs has gained popularity [8]. Under this approach, cells are prepared in good manufacturing practice (GMP) facilities and delivered to patients. Although in its infancy due to regulatory and technical challenges and costs, pioneeristic clinical trials showed positive outcomes [9]. Nevertheless, several variables in the published studies may interfere with the analysis of the results, and more robust clinical trials are necessary for generating reliable evidence with which to support these treatments. In this frame, two major points have to be considered and sifted. The source of MSCs, since several sources of autologous or allogeneic cells were used in the clinical trials, and are very often underestimated, the culturing conditions, especially for medium supplements, span from fetal bovine serum (FBS) or human platelet lysate (hPL)/serum (hS) to the recently GMP-approved serum-free (SF) and xeno-free (XF) components to obtain serum/xeno-free media (SFM/XFM). The main advantage of SFM/XFM formulations is that the media have only synthetic, recombinant, or human-derived purified substances without the addition of serum as a supplement.

The conscious choice of the medium, based on reliable data, is of great relevance since it clearly emerged in in vitro and preclinical reports that culturing conditions may greatly affect MSCs phenotype and molecular fingerprint [10,11,12,13]. In this perspective, more work is needed to compare cells expanded under different supplements, especially for new generation SFM/XFM that, albeit maintaining the basic differentiation potential and surface marker expression profile characteristic of MSCs [14], are still poorly characterized. Therefore, molecular pillars, such as reliable housekeeping genes (HKGs), are required to directly evaluate those expansion methodologies side by side.

The aim of this work is, therefore, to analyze the stability of five commonly used HKGs in adipose-derived MSCs (ASCs) cultivated under either FBS as standard condition or two commercially available SFM/XFM. A comprehensive analysis was performed with four applets recognized by the scientific community to identify the most stable candidates. Eventually, a final stability ranking summarizing single applet outcomes is provided for the best HKGs and results used to test amount of genes (*CCL5*, *IL6* and *LIF*) related to MSCs’ therapeutic potential for OA treatment.

## 2. Materials and Methods

### 2.1. Ethics

The study was performed under Informed Consent administration to patients and Institutional Review Board approval (San Raffaele Hospital Ethics Committee approval on date 16 December 2020, registered under number 214/int/2020). The study followed the 1964 Helsinki declaration and its later amendments or comparable ethical standards.

### 2.2. Adipose Mesenchymal Stromal Cells (ASCs) Isolation and Culture

Waste adipose tissue of four female donors (median 37 years old) undergoing elective plastic surgery was collected and digested with 0.075% *w*/*v* type I collagenase (Worthington Biochemical Co., Lakewood, NJ, USA) for 30 min at 37 °C. Digested tissue was filtered with a 100 µm cell strainer. The flow-through was centrifuged (1000× *g*, 5 min) to recover cells that were seeded at 10 × 10^3^ cells/cm^2^ and cultivated at 37 °C, 5% CO_2_, and 95% humidity up to passage 1. TrypLE™ Express Enzyme (animal origin-free, recombinant enzyme; ThermoFisher, Waltham, MA, USA) was used to dissociate cells. For all steps, CTS™ DPBS with calcium and magnesium, manufactured in state-of-the-art cGMP (ThermoFisher) was used. Three different conditions were set:

(i) DMEM/F12 + 10% FBS (GE Healthcare, Piscataway, NJ, USA), 1% L-glutamine plus penicillin–streptomycin (PSG) (Life Technologies, Carlsbad, CA, USA). This condition will be named hereafter (F);
(ii) StemPro™ MSC SFM XenoFree (xeno-free, cGMP compliant) (ThermoFisher), 1% PSG. Before seeding, flasks were coated with CELLstart™ Substrate (xeno-free, cGMP compliant) (ThermoFisher) as per manufacturer’s instruction to enhance cell adhesion and growth in absence of serum. Condition is named (X1);
(iii) StemFit^®^ For Mesenchymal Stem Cells (xeno-free) (Amsbio, Cambridge, MA, USA), 1% PSG. Before seeding, flasks were coated with iMatrix-511 expressed in CHO cells for easier translation into GMP (Amsbio) as per manufacturer’s instruction to enhance cell adhesion and growth in absence of serum. iMatrix-511 is comprised of recombinant Laminin-511 E8 protein fragments. Condition is named (X2).

### 2.3. ASCs Immunophenotype by Flow Cytometry

ASCs at passage 1 were analyzed by flow cytometry to score the expression of MSC (CD73-PE clone REA804, CD90-FITC clone REA897; Miltenyi Biotec, Bergisch Gladbach, Germany) and hemato-endothelial (CD45-PE Vio770 clone REA747; Miltenyi Biotec. CD31-APC clone WM59; Biolegend, San Diego, CA, USA) markers. A minimum of 30,000 events were acquired with a CytoFLEX flow cytometer (Beckman Coulter, Fullerton, CA, USA).

### 2.4. RNA Extraction and mRNA Profiling

RNA was isolated from cells at passage 1 with the miRNeasy Micro Kit (Qiagen, Hilden, Germany) following the manufacturer’s protocol. After quantification, equal amounts of purified RNA for each sample were reverse transcribed with the iScript™ cDNA Synthesis Kit (BioRad, Hercules, CA, USA) and preamplified with SsoAdvanced™ PreAmp Supermix (BioRad), both following manufacturer’s instructions. Amplifications were carried out with iTaq Universal SYBR Green Supermix (BioRad) in a CFX Opus Real-Time PCR Systems (BioRad) as per manufacturer’s protocol. The following HKG were tested: *ACTB* (Actin Beta), *EF1A* (Eukaryotic Translation Elongation Factor 1 Alpha 1), *RPLP0* (Ribosomal Protein Lateral Stalk Subunit P0), *TBP* (TATA-Box Binding Protein), and *GAPDH* (Glyceraldehyde-3-Phosphate Dehydrogenase). The following MSC-specific genes were assayed: *LIF* (Leukemia Inhibitory Factor), *CCL5* (C-C Motif Chemokine Ligand 5), and *IL6* (Interleukin 6). Primer sequences will be provided upon request.

### 2.5. Data Analysis

HKGs stability was tested with four algorithms: NormFinder [15], geNorm [16], comparative ΔCt method [17], and BestKeeper [18]. HKGs candidates’ stability is evaluated by each algorithm with different variables. Normfinder relies on linear scale quantitative data and allows the definition of a stability value (low value for high stability). geNorm provides an M-value based on the average pairwise expression ratio, with stability being defined by M < 1.5. In the ΔCt approach, “pairs of genes” are compared. Standard deviation (SD) is the base for BestKeeper, with a higher value indicating low stability. Each approach generated an HKG stability ranking, with a series of continuous integers starting from 1. The four rankings were computed by RefFinder, a web-based comprehensive tool that assigns an appropriate weight to an individual HKG calculating the geometric mean of the different rankings to generate the overall final ranking [19].

### 2.6. Hierarchical Clustering and Principal Component Analysis

Hierarchical clustering and principal component analysis (PCA) of the Ct values were obtained with the ClustVis [20] webtool (https://bio.tools/clustvis). Raw Ct values were loaded from an Excel sheet, allowing the software to detect table delimiter and column and row annotations. Afterwards, in the data preprocessing options page, the following setup was selected: no transformation, row centering, no row scaling, and SVD with imputation as PCA method. For the heat map, the following parameters were followed: correlation as clustering distance for rows and columns, average as clustering method for rows and columns, and tighter cluster first for tree ordering for rows and columns.

### 2.7. Statistical Analyses

Statistical analyses were performed with GraphPad Prism Software (version 8.0.2, GraphPad, San Diego, CA, USA) and the stability algorithm NormFinder, geNorm, comparative ΔCt method, and BestKeeper. For comparison of gene expression data between conditions, a one-sample t-test was performed on fold changes with hypothetical mean value set at 1. The level of significance was set at *p*-value ≤ 0.05.

## 3. Results

### 3.1. ASCs Characterization

**Figure 1:**
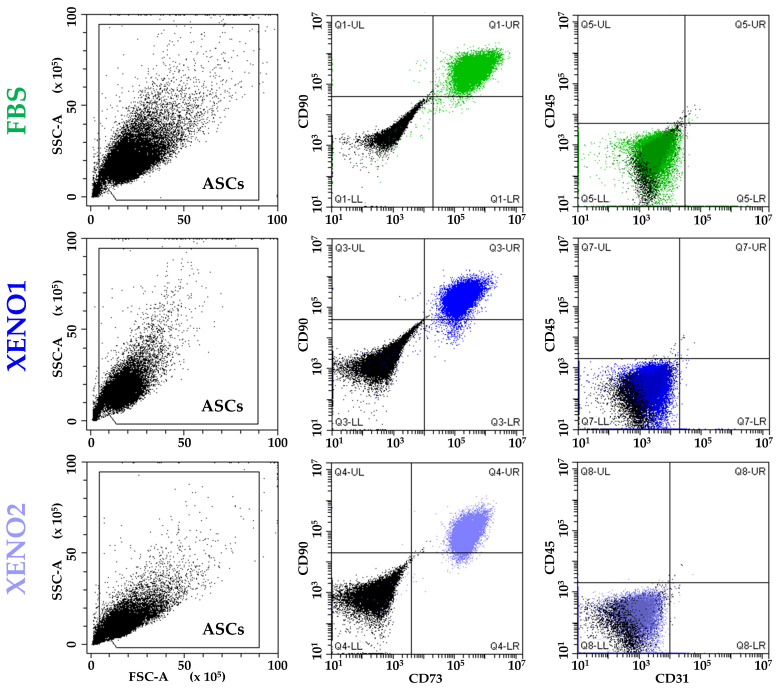
ASCs immunophenotype. ASCs cultured in standard condition (FBS) or serum/xeno-free media (XENO1/2) resulted positive to MSCs markers CD73 and CD90 and negative to haemato-endothelial markers CD45 and CD31. Plots from a representative donor are shown.

ASCs cultured in the three different conditions (FBS, and SFM/XFM named Xeno1 and Xeno2) confirmed the presence of standard MSC markers (CD73 and CD90) and the absence of haemato-endothelial epitopes (CD45 and CD31) (Figure 1).

### 3.2. Candidate HKGs Expression

**Figure 2:**
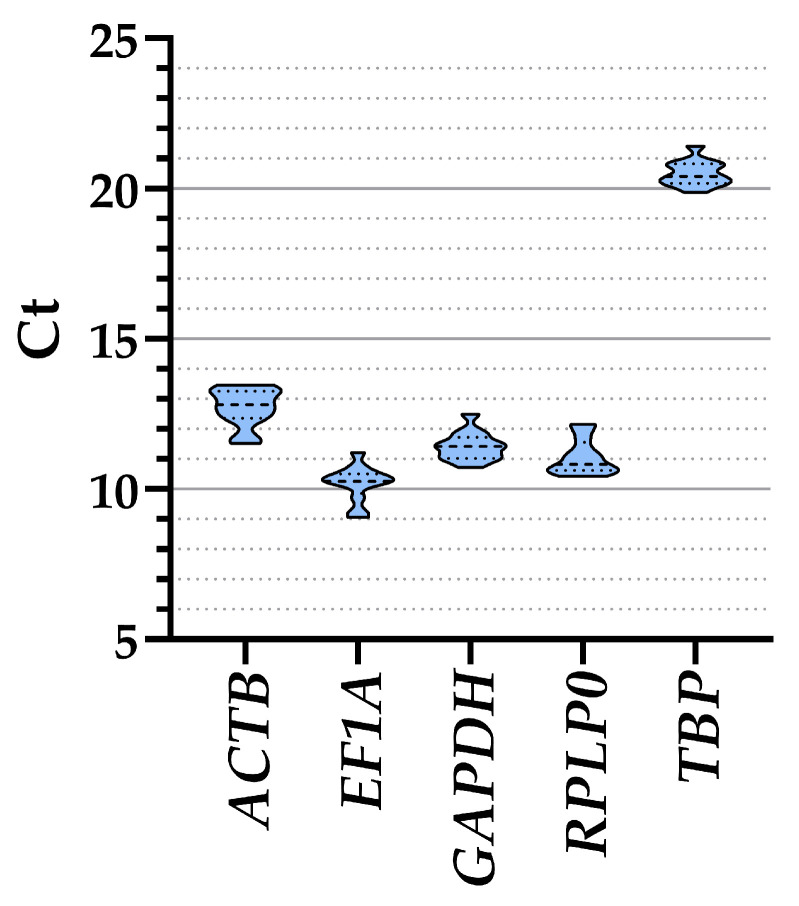
Ct values of the five HKGs across all conditions. Violin plots are shown, with dashed-line pattern for median and dotted-line for quartiles.

The analysis of all ASCs samples together, regardless of the culture media, showed that *EF1A* had the lower Ct values and therefore higher amount (10.19 ± 0.17, mean ± SEM, n = 12), followed by *RPLP0* (11.07 ± 0.17), *GAPDH* (11.42 ± 0.13), *ACTB* (12.72 ± 0.18), and, eventually, *TBP* (20.51 ± 0.12) (Table 1 and Figure 2). Taking into consideration the separate conditions, a similar range of expression emerged for all genes (≤1 C_t_ value between treatments) (Table 1), confirming their suitability as HKGs. To ensure that gene expression of the five genes was not influenced by co-regulation given by proximity on the same chromosome, a gene location search was performed. *ACTB* is on chromosome 7, *EF1A* and *TBP* are on chromosome 6, with a distance of approximately 100 million bases (*TBP* at the final extremity of the chromosome), while *GAPDH* and *RPLP0* are on the extremities of chromosome 12, separated by >100 million bases and at the two opposite sides of the centrosome. With >10 million bases being a stringent discriminant for expression co-regulation [21], the five HKGs under analysis can be considered independent in their basal transcription values.

**Figure 3:**
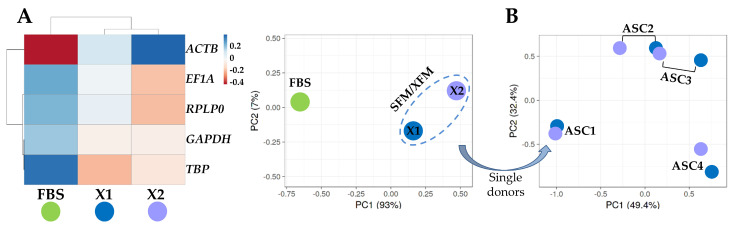
Heat map and principal component analysis (PCA) of HGKs expression values. (**A**) Heat map and PCA of samples divided by FBS or SFM/XFM culturing conditions. Each condition is the result of the merged 4 donors. In the heat map, negative values mean lower Ct (higher amount), while positive values mean higher Ct (lower amount) with respect to mean values after row centering for each HGKs. Both rows and columns were clustered using correlation distance and average linkage. No transformation and no scaling were applied for the dataset. In PCA, the *X*- and *Y*-axis show principal component 1 and principal component 2, which explain 93% and 7% of the total variance, respectively. (**B**) PCA of Ct values of 8 samples (4 ASCs, each in the 2 different SFM/XFM media) cultured in absence of serum. The *X*- and *Y*-axis show principal component 1 and principal component 2, which explain 49.4% and 39.2% of the total variance, respectively.

To identify an overall influence of the culture conditions, either FBS-containing or SFM/XFM, on the expression trend of the five analyzed HKGs, a hierarchical clustering and principal component analysis (PCA) were performed on the Ct values under the different media of each gene (Figure 3A). There was a clear emergence of a sharp dichotomy between FBS-containing medium and the SFM/XFM samples that clustered together. To further dissect the effect of the chemically defined media, a deeper PCA was run with all eight samples cultivated in absence of FBS (Figure 3B). The plot showed that a donor effect prevailed, with the samples of the same donor in the two different synthetic media clustering together. Overall, these data suggest that SFM/XFM are able to homogeneously drive HKGs signature, maintaining a donor-dependent molecular signature.

### 3.3. Analysis of HKGs Stability

To determine the reliability of the five HKGs, four applets were used and the final stability ranking was generated (Table 2). Considering the media separately, for ASCs in FBS the most stable HKG resulted to be *EF1A* (geomean of the four applets’ rankings = 1.19) followed by *TBP* (1.86), while *GAPDH* was the worst performer (4.73). Notably, the order was greatly changed in the SFM/XFM media. In fact, for both synthetic formulations, *GAPDH* resulted as the best candidate (1.00 for X1 and 1.32 for X2), followed by *TBP* (1.86 for X1 and 2.00 for X2). *EF1A* dropped at the bottom of the rankings, being pre-last for X1 (4.00; *RPLP0* last with 5.00) and last for X2 (5.00; *RPLP0* pre-last 2.83). The similar behavior of the two SFM/XFM media was confirmed by the ranking obtained analyzing the two conditions together, which confirmed *GAPDH* in the first position (1.00), followed by *TBP* (1.68), and *EF1A* (4.23)/*RPLP0* (4.73) last.

Further, FBS/X1 and FBS/X2 analyses were performed to allow comparison between standard FBS-cultured samples and those in the new generation synthetic media (Table 2). In the wake of similar behavior for the SFM/XFM media, for both comparisons, *EF1A* and *TBP* were at top of the rankings (FBS/X1: *EF1A* 1.32 and *TBP* 1.41; FBS/X2: *TBP* 1.00 and *EF1A* 2.00), while *RPLP0* and *ACTB* were at the bottom (FBS/X1: *RPLP0* 4.00 and *ACTB* 5.00; FBS/X2: *RPLP0* 3.22 and *ACTB* 5.00). Eventually, all three conditions were analyzed together (ALL in Table 2). *TBP* (1.41) and *GAPDH* (1.57) ranked first and second, respectively. *RPLP0* (4.00) and *ACTB* (5.00) resulted pre-last and last.

Finally, HKGs stability for each donor across the three conditions was tested (Table 3). Of note, the rankings resulted to be donor-specific. *TBP* was the best for ASC1 (1.73) and ASC3 (1.19), while second for ASC2 (2.00) and last for ASC4 (5.00). *GAPDH* was the top for ASC2 (1.73), fourth for ASC1 (2.99), and second for ASC3 (1.41) and ASC4 (1.73). *ACTB* was the most stable for ASC4 (1.32), while it was last for the other donors (ASC1: 3.98; ASC2: 5.00; ASC3: 5.00).

### 3.4. Effect of HKGs Choice on Target Gene Expression Evaluation

**Figure 4:**
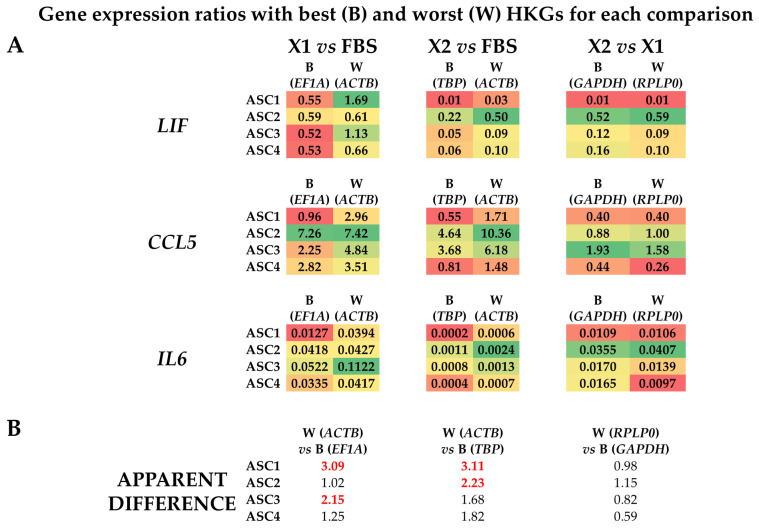
Effect of suboptimal HKGs choice on gene expression evaluation for each condition at single donor level. (**A**) The amount of *LIF*, *CCL5*, and *IL6* was compared using the best (B) or worst (W) HKG for each analyzed couple, as per Table 2. Color code indicates a gradient between the lowest (red) and highest (green) ratios for each comparison. (**B**) Apparent differences in gene expression ratios for each ASC donor using worst (W) vs. best (B) HKGs. In red and bold are values ≥ 2.

**Figure 5:**
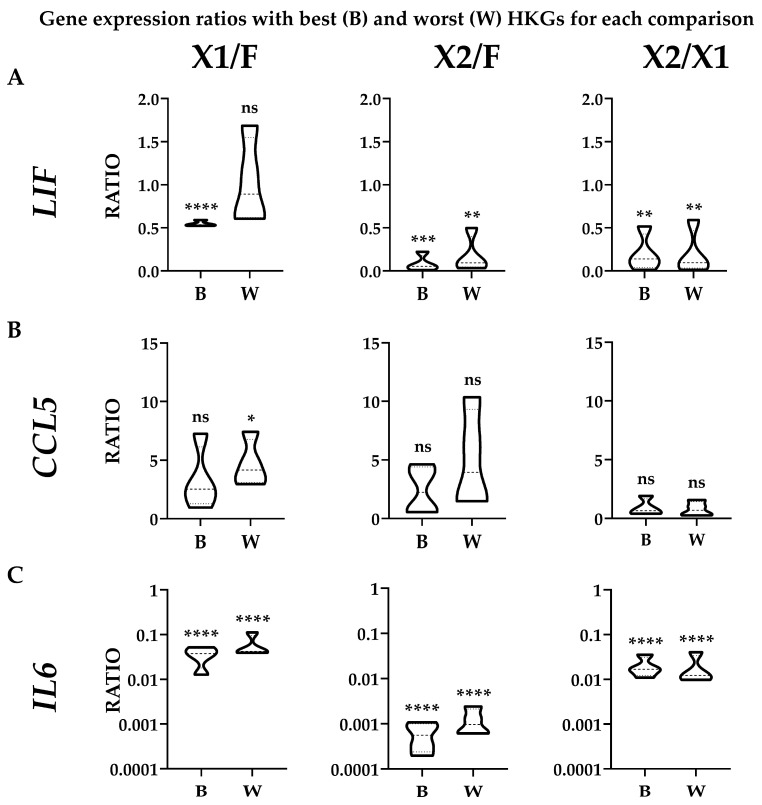
Effect of suboptimal HKGs choice on gene expression evaluation for each condition with donors’ results merged. The amount of *LIF* (panel (**A**)), *CCL5* (panel (**B**)), and *IL6* (panel (**C**)) was compared using the best (B) or worst (W) HKG for each analyzed couple, as per Table 2. Single donor values were merged to obtain expression range for each condition. N = 4; ns stands for not significant; * stands for *p*-value ≤ 0.05; ** stands for *p*-value ≤ 0.01; *** stands for *p*-value ≤ 0.001; **** stands for *p*-value ≤ 0.0001.

To evaluate the effect of suboptimal HKGs selection on gene expression, three genes (*LIF, CCL5*, and *IL6*) whose soluble products are involved in OA, and therefore monitored in their amounts for therapeutic approaches, were analyzed in ASCs comparing their expression levels in the different media with best (B) or worst (W) reference genes identified in Table 2 (Figure 4A,B). With a focus on single donors, for X1 vs. FBS, the top HKG (*EF1A*) showed a constant reduction for both *LIF* and *IL6* when using the synthetic medium in all donors, with an increase for *CCL5* except for donor 1. The use of the less reliable HKG (*ACTB*) led to an apparent higher ratio (W/B ≥ 2) for ASC1 (3.09) and ASC3 (2.15), resulting, for these donors, in an absence of modulation or false increase for *LIF* and upregulation for *CCL5* in ASC1. For X2 vs. FBS, with the most reliable HKG (*TBP*), the reduction in *LIF* and *IL6* in all ASCs in SFM/XFM was confirmed, as well as a less defined and donor-dependent situation for *CCL5* (increase in ASC2/3 and reduction in ASC1/4). The unstable *ACTB* led to an apparent higher ratio (W/B ≥ 2) for ASC1 (3.11) and again ASC2 (2.23), with ASC3/4 having a similar but less pronounced (W/B < 2) trend. This led to a false upregulation for ASC1/4 for *CCL5*. Eventually, for X2 vs. X1, *LIF* and *IL6* resulted as downregulated in X2 in all donors when analyzed with the optimal *GAPDH*, as well as *CCL5*, with the exception of ASC3. The closer behavior of HKGs performance, either top or bottom of the ranking, for the two synthetic media was confirmed by the high similarity observed with the worst HKG (*RPLP0*). Eventually, statistics were performed on donors’ data gathered together (Figure 5) to score gene expression modulation more strictly related to the culturing conditions and trying to avoid the bias of single donor variability. For *LIF*, the reduced amount observed with the top normalizers in both SFM/XFM was lost for X1/F and reduced in significance for X2/F using the less reliable HKG. For *CCL5*, at condition level, no differences emerged in all comparison with best HKG, while suboptimal choice led to an apparent increase in X1 vs. F. For *IL6*, the reduction in both synthetic media clearly emerged, with its strength avoiding loss of significance with the worst HKG. Overall, comparing conditions, the use of less performant HKGs ended up in an apparent 1.9-fold increase (W/B) for X1 vs. F and 2.2-fold for X2 vs. F, while, again, a very stable situation (0.9-fold) characterized X2 vs. X1. As a summary, whether for X1/F and X2/F comparisons, the selection of the HKG affected the outcomes or their significance at both donor and overall levels; for X1/X2, comparable results were always obtained with both normalizers, confirming the higher HKG performance similarity for cells in synthetic media. These data suggest the crucial importance of HKG selection when different media are analyzed side by side.

## 4. Discussion

This work is, to our knowledge, the first to report a selection of reliable HKGs for adipose-derived MSCs cultivated under SFM/XFM conditions. These results will pave the way for similar studies with other MSC types or new-generation synthetic media that will be available for clinical expansion in the next years and are envisioned as the future of clinically relevant GMP-grade products.

MSCs for clinical applications are classified as advanced therapy medicinal products (ATMPs) and good manufacturing practices (GMP) have to be followed for their expansion to ensure consistent production and quality standards following European Regulation 1394/2007/EC and Directive 2009/120/EC [22]. A fundamental pillar to obtain GMP-grade MSCs is the cultivation process. In the wake of basic research protocols, MSCs are traditionally expanded in a chemically defined media, such as several versions of modified Eagle’s medium (MEM), supplemented with animal serum, with the most widely used being fetal bovine serum (FBS). However, FBS is not a sustainable option for GMP products since its composition is not well defined, and, even more, it presents a significant risk of interspecies cross-contamination [23]. To reduce these issues, alternatives are now available, including human platelet lysate (hPL) [24]. Nevertheless, batch-to-batch variations persist, alongside the potential risk of disease transmission and, most importantly with respect to FBS, a limited availability, therefore representing bottlenecks for large-scale GMP production. For these reasons, new, chemically defined synthetic serum/xenogeneic-free (SFM/XFM) media were recently released on the market for MSC expansion. Some of them are already GMP-compliant, such as the first one used in this study (Xeno1), or with a GMP-grade option in preparation, such as the second one (Xeno2). Cells expanded in these media showed typical MSCs morphology, surface markers, multipotent differentiation capability, and immunomodulatory capacity [14], with increased proliferation rates compared to FBS-containing media [12,14]. Despite these promising results and enhanced batch-to-batch consistency in the cell manufacturing process, pioneeristic comparative reports showed how these new formulations may promote differences in MSC performance [25,26,27]. Therefore, more studies are needed to deeply dissect the features of MSCs expanded in SFM/XFM, since for classic media it was reported how differences in culturing conditions may deeply affect their phenotypic and molecular signature [10,28,29] and clinical relevance [30].

In this report, to facilitate future molecular analyses of MSCs cultivated under SFM/XFM, a stability analysis on putative housekeeping genes (HKGs) was performed on adipose-derived MSCs (ASCs) and compared with cells grown in standard FBS-containing conditions. This issue is of particular relevance since different media may impact not only the overall molecular signature of MSCs but also the stability of HKGs [31,32,33,34], thus potentially overestimating or underestimating genes and pathways of interest for a specific clinical application. Under these premises, *EF1A* and *GAPDH* inverted their positions in the stability rankings based on the analyzed media (Table 2). *EF1A* was the most stable for FBS and last or pre-last for SFM/XFM, while *GAPDH* behaved oppositely. It should be noted that the two synthetic media had an almost superimposable pattern of HKGs reliability, confirmed by the identical positions in the coupled (X1/X2) analysis. This reinforces the notion that, although without details on proprietary media formulation, similar conditions may confer a comparable molecular signature to cells facilitating future works on SFM/XFM products, even if different from those used herein. For these reasons, a direct testing of MSCs under different media using stable HKGs results is mandatory to avoid the use of low-performance normalizers borrowed from similar, albeit different, analyses. Accordingly, in this work, *EF1A*, the poorly stable HKGs for SFM/FM, fell among the two most stable ones (with *TBP*) for both FBS/X1 and FBS/X2. To further confirm that each multiple comparison must be performed with the best HKG, the triple FBS/X1/X2 combination showed *TBP* to be the best reference gene, with SFM/XFM-specific *GAPDH* in second position and FBS-specific *EF1A* in third. Overall, our data confirm how each condition and each comparison should be carefully dissected for best reliability.

Together with a deep analysis for each culture condition, another issue for clinical application of MSCs is the identification of the physiological and molecular features of each donor. In fact, the idea for GMP-expanded MSCs is to create an off-the-shelf bank with deeply characterized batches, as proposed for COVID-19 patients [35]. This choice comes in the wake of the personalized medicine approach [36], where a direct link between the profile of the patient and the features of the therapeutics is mandatory. In this frame, our group already showed how only a proper normalization strategy may reliably identify the differences between MSC donors for their musculoskeletal-diseases-related miRNA portfolio in extracellular vesicles isolates to be used as off-the-shelf therapeutics [37]. Consistently, in this work, it clearly emerged how, congruent to culturing conditions, the four donors also have different HKGs stability fingerprint (Table 3). In fact, if ASC2 and 3 resulted as similar, with *TBP* and *GAPDH* at top of the ranking and *ACTB* at the bottom, ASC1 still had *TBP* in first position but *GAPDH* in pre-last just before *ACTB*, while ASC4 was completely reversed with *ACTB* and *GAPDH* as best candidates and *TBP* the worst performer. Thus, identification of the most appropriate HKG will be mandatory for single isolates banked for personalized medicine approaches.

Eventually, the effect of suboptimal HKGs choice clearly emerged by direct comparison of gene expression amount for *LIF*, *CCL5*, and *IL6* between conditions. *LIF* (Leukemia Inhibitory Factor) is a pleiotropic cytokine belonging to the interleukin-6 superfamily [38]. For MSCs, this cytokine was shown to be involved with their proangiogenic potential [39]. Moreover, LIF is produced by joint tissue cells and is overexpressed in osteoarthritis (OA) [40], playing an important role in its pathogenesis [41]. For these reasons, MSCs type or donor with reduced LIF would be preferable as OA therapeutics. In this perspective, both SFM/XFM showed reduced expression, with X2 having the lowest values (especially for ASC1/3/4). The use of the less stable HKGs alters the reliable quantification of their expression, mainly for ASC1/3 when X1 or X2 and FBS are compared. *CCL5* (C-C Motif Chemokine Ligand 5) is an inflammatory chemokine and potent chemoattractant [42]. In MSCs, this gene is strongly upregulated in inflammatory conditions [43], similar to those encountered after administration to the diseased joint [44]. Also, CCL5 was found to be upregulated in OA synovial fluid [45]. Therefore, again, lower CCL5 levels might be beneficial for therapeutic MSCs. Opposite to *LIF*, *CCL5* resulted as upregulated in several donors in SFM/XFM, although with a less clear picture using the best HKG, where ASC1 had comparable (X1 vs. FBS) or lower levels (X2 vs. FBS) as well as ASC4 (X2 vs. FBS). Of note, the worst HKG did not allow the detection of differences in gene modulation between donors that always resulted as upregulated for both synthetic media. Finally, *IL6* (Interleukin 6) is an inflammatory cytokine [46]. It is involved in joint inflammation and found to be upregulated in synovial fluid from OA patients, correlating with disease incidence and severity [47]. As for the other analyzed factors, lower levels may be beneficial for MSCs when used for joint diseases. Both SFM and XFM had a clear trend of expression reduction (up to 5000-fold for ASC1 in X2 vs. FBS). Due to the force of downregulation, the use of the less stable HKG did not result in false gene expression evaluation, albeit reduction was underestimated. Overall, these data demonstrate that wrong evaluation of gene amount caused by suboptimal HKG can occur in case of reduced modulation, and this is of relevance when several donors are compared to choose the most promising for a specific pathology. It should be noted that this effect at the donor level was mitigated when each condition was analyzed as a whole, merging donor data. In fact, the use of less performant HKG changed gene expression comparison in the X1/F for both *LIF*, which passed from downregulation in the SFM/XFM to no modulation, and *CCL5*, which passed from no modulation in the SFM/XFM to upregulation. For the other comparisons, a loss of one point of significance was observed for *LIF* in X2/F. These variations were due to a misleading twofold increase for X1/F and X2/F given by less performant HKG. Taking all these data together, the higher variations with worst HKGs observed for X1 or X2 vs. FBS with respect to those emerged for X1 vs. X2 confirmed how optimal reference gene choice is more relevant when comparing diverging conditions such as synthetic versus serum supplements, while similar media have comparable HKG performance.

We are aware that this study has some limitations. The number of SFM/XFM media available on the market is rapidly increasing with some of those now released as GMP-compliant products. Nevertheless, the very similar HKGs performance for the two fully synthetic media used in this study may lead us to speculate on a similar behavior for other SFM/XFM formulations. Also, a higher number of HKGs have been proposed through the years. We opted for a selection among those more popular and often available in many laboratories and research centers. In fact, considering the category of HKGs consisting of genes that are involved in the regulation of basic and ubiquitous cellular functions required for the survival of most cell types [48], *GAPDH* and *ACTB* were selected. *TBP* was analyzed due to its performance in adipose MSCs, starting from pioneeristic works to identify stable HKGs [49]. *EF1A* and *RPLP0* resulted as the genes with the most stable expression in fibroblasts from non-OA or OA patients [50], making them interesting candidates to be scored for cells involved in OA-related therapies. Lastly, as further suggested by our data on donor-related stability, extreme care should be taken when selecting the most appropriate HKGs to be used to fingerprint the batches under study or for clinical purposes. When considering a new research strategy or clinical application, if not present in the literature for the intended purposes, a proper HKG validation might be considered as the initial step of a wider process. The initial screening given by molecular fingerprint has to be intended as a trailblazer for other tests, such as to confirm gene expression modulation at the protein level and the importance of these modulations in a wider scenario given by the interaction with the environment and the target cells and tissues. Thus, the analysis on the limited number of ASCs and HKGs herein analyzed must be envisioned as a proof of concept for SFM/XFM studies on ASCs and to stimulate researchers to consider HKGs stability analysis as an initial step, if feasible, within the frame of their own needs.

## 5. Conclusions

This work has the aim to propose reliable HKGs for adipose-derived MSCs cultivated under fully synthetic media for translational approaches in regenerative medicine for OA, and more in general for pathologies where MSCs are envisioned as next-generation therapeutics. It clearly emerged how both culturing conditions and donors may greatly affect HKGs reliability, therefore being an incentive to researchers to test, if feasible, the best candidate for their experimental or clinical needs.

## Figures and Tables

**Table 1:** Mean Ct values of HKGs analyzed in the study.

	*ACTB*	*EF1A*	*GAPDH*	*RPLP0*	*TBP*
ALL	12.72 ± 0.18	10.19 ± 0.17	11.42 ± 0.13	11.07 ± 0.17	20.51 ± 0.12
FBS	12.28 ± 0.39	10.42 ± 0.27	11.59 ± 0.34	11.27 ± 0.29	20.85 ± 0.18
XENO1	12.80 ± 0.16	10.18 ± 0.29	11.35 ± 0.10	11.08 ± 0.30	20.28 ± 0.17
XENO2	13.09 ± 0.15	9.98 ± 0.27	11.34 ± 0.16	10.85 ± 0.24	20.39 ± 0.13

(Mean ± SEM, n = 12 for “ALL” and n = 4 for “FBS, XENO1, and XENO2”).

**Table 2:** Stability rankings of tested HKGs across the different media separately or combination of media.

Cond	Geomean		Delta CT		BestKeeper		NormFinder		Genorm	
**FBS**	*EF1A*	1.19	*EF1A*	0.33	*TBP*	0.30	*EF1A*	0.08	*EF1A|RPLP0*	0.17
	*TBP*	1.86	*TBP*	0.40	*EF1A*	0.43	*TBP*	0.20		
	*RPLP0*	2.28	*RPLP0*	0.41	*RPLP0*	0.45	*RPLP0*	0.28	*TBP*	0.23
	*ACTB*	4.23	*ACTB*	0.48	*GAPDH*	0.62	*ACTB*	0.36	*ACTB*	0.32
	*GAPDH*	4.73	*GAPDH*	0.66	*ACTB*	0.67	*GAPDH*	0.62	*GAPDH*	0.46
**X1**	*GAPDH*	1.00	*GAPDH*	0.43	*GAPDH*	0.17	*GAPDH*	0.13	*TBP|GAPDH*	0.26
	*TBP*	1.86	*TBP*	0.46	*ACTB*	0.24	*TBP*	0.23		
	*ACTB*	2.71	*ACTB*	0.55	*TBP*	0.28	*ACTB*	0.38	*ACTB*	0.34
	*EF1A*	4.00	*EF1A*	0.60	*EF1A*	0.47	*EF1A*	0.48	*EF1A*	0.43
	*RPLP0*	5.00	*RPLP0*	0.75	*RPLP0*	0.50	*RPLP0*	0.68	*RPLP0*	0.56
**X2**	*GAPDH*	1.32	*GAPDH*	0.44	*TBP*	0.22	*GAPDH*	0.14	*RPLP0|GAPDH*	0.29
	*TBP*	2.00	*TBP*	0.55	*ACTB*	0.24	*TBP*	0.36		
	*ACTB*	2.71	*ACTB*	0.57	*GAPDH*	0.30	*ACTB*	0.43	*ACTB*	0.43
	*RPLP0*	2.83	*RPLP0*	0.59	*RPLP0*	0.41	*RPLP0*	0.48	*TBP*	0.49
	*EF1A*	5.00	*EF1A*	0.67	*EF1A*	0.46	*EF1A*	0.58	*EF1A*	0.56
**X1/X2**	*GAPDH*	1.00	*GAPDH*	0.43	*GAPDH*	0.23	*GAPDH*	0.18	*TBP|GAPDH*	0.36
	*TBP*	1.68	*TBP*	0.49	*TBP*	0.25	*TBP*	0.28		
	*ACTB*	3.00	*ACTB*	0.56	*ACTB*	0.30	*ACTB*	0.42	*ACTB*	0.39
	*EF1A*	4.23	*EF1A*	0.61	*RPLP0*	0.46	*EF1A*	0.51	*EF1A*	0.48
	*RPLP0*	4.73	*RPLP0*	0.65	*EF1A*	0.47	*RPLP0*	0.56	*RPLP0*	0.55
**FBS/X1**	*EF1A*	1.32	*EF1A*	0.51	*TBP*	0.37	*EF1A*	0.29	*EF1A|TBP*	0.33
	*TBP*	1.41	*TBP*	0.53	*GAPDH*	0.39	*TBP*	0.33		
	*GAPDH*	2.71	*GAPDH*	0.56	*EF1A*	0.43	*GAPDH*	0.38	*GAPDH*	0.43
	*RPLP0*	4.00	*RPLP0*	0.61	*RPLP0*	0.49	*RPLP0*	0.47	*RPLP0*	0.52
	*ACTB*	5.00	*ACTB*	0.67	*ACTB*	0.55	*ACTB*	0.56	*ACTB*	0.58
**FBS/X2**	*TBP*	1.00	*TBP*	0.56	*TBP*	0.32	*TBP*	0.30	*EF1A|TBP*	0.38
	*EF1A*	2.00	*EF1A*	0.58	*EF1A*	0.40	*GAPDH*	0.37		
	*GAPDH*	3.13	*RPLP0*	0.59	*GAPDH*	0.46	*RPLP0*	0.37	*RPLP0*	0.47
	*RPLP0*	3.22	*GAPDH*	0.59	*RPLP0*	0.48	*EF1A*	0.38	*GAPDH*	0.50
	*ACTB*	5.00	*ACTB*	0.82	*ACTB*	0.62	*ACTB*	0.74	*ACTB*	0.63
**ALL**	*TBP*	1.41	*GAPDH*	0.54	*TBP*	0.34	*GAPDH*	0.28	*EF1A|TBP*	0.39
	*GAPDH*	1.57	*TBP*	0.54	*GAPDH*	0.36	*TBP*	0.31		
	*EF1A*	2.28	*EF1A*	0.57	*EF1A*	0.43	*EF1A*	0.39	*GAPDH*	0.45
	*RPLP0*	4.00	*RPLP0*	0.63	*RPLP0*	0.48	*RPLP0*	0.46	*RPLP0*	0.52
	*ACTB*	5.00	*ACTB*	0.73	*ACTB*	0.50	*ACTB*	0.63	*ACTB*	0.60

Cond stands for condition. FBS stands for ASCs cultured in FBS-containing medium. X1 stands for ASCs cultivated in SFM/XFM XENO1 and X2 for XENO2. ALL stands for all conditions analyzed together.

**Table 3:** Stability rankings of tested HKGs across the different ASC-donors separately.

Cond	Geomean		Delta CT		BestKeeper		NormFinder		Genorm	
**ASC1**	*TBP*	1.73	*TBP*	0.47	*RPLP0*	0.20	*TBP*	0.13	*EF1A|GAPDH*	0.14
	*RPLP0*	2.00	*RPLP0*	0.52	*ACTB*	0.35	*RPLP0*	0.13		
	*EF1A*	2.45	*EF1A*	0.56	*TBP*	0.37	*EF1A*	0.44	*TBP*	0.30
	*GAPDH*	2.99	*GAPDH*	0.59	*EF1A*	0.59	*GAPDH*	0.50	*RPLP0*	0.38
	*ACTB*	3.98	*ACTB*	1.01	*GAPDH*	0.65	*ACTB*	1.00	*ACTB*	0.63
**ASC2**	*GAPDH*	1.73	*GAPDH*	0.32	*TBP*	0.05	*GAPDH*	0.11	*RPLP0|TBP*	0.13
	*TBP*	2.00	*EF1A*	0.33	*RPLP0*	0.09	*EF1A*	0.15		
	*RPLP0*	2.06	*RPLP0*	0.38	*GAPDH*	0.24	*RPLP0*	0.30	*GAPDH*	0.29
	*EF1A*	2.83	*TBP*	0.40	*EF1A*	0.29	*TBP*	0.33	*EF1A*	0.31
	*ACTB*	5.00	*ACTB*	0.50	*ACTB*	0.42	*ACTB*	0.46	*ACTB*	0.39
**ASC3**	*TBP*	1.19	*GAPDH*	0.35	*TBP*	0.07	*TBP*	0.04	*TBP|GAPDH*	0.08
	*GAPDH*	1.41	*TBP*	0.37	*GAPDH*	0.13	*GAPDH*	0.04		
	*EF1A*	3.00	*EF1A*	0.38	*EF1A*	0.19	*EF1A*	0.08	*EF1A*	0.15
	*RPLP0*	4.00	*RPLP0*	0.59	*RPLP0*	0.21	*RPLP0*	0.55	*RPLP0*	0.27
	*ACTB*	5.00	*ACTB*	0.86	*ACTB*	0.70	*ACTB*	0.85	*ACTB*	0.51
**ASC4**	*ACTB*	1.32	*ACTB*	0.32	*GAPDH*	0.17	*ACTB*	0.07	*ACTB|GAPDH*	0.15
	*GAPDH*	1.73	*EF1A*	0.34	*RPLP0*	0.21	*EF1A*	0.16		
	*EF1A*	2.63	*GAPDH*	0.39	*ACTB*	0.24	*GAPDH*	0.30	*EF1A*	0.29
	*RPLP0*	3.36	*RPLP0*	0.45	*EF1A*	0.44	*RPLP0*	0.38	*RPLP0*	0.35
	*TBP*	5.00	*TBP*	0.46	*TBP*	0.57	*TBP*	0.42	*TBP*	0.39

Cond stands for condition.

## Data Availability

Raw data for this study are available at https://osf.io/ea3y7/?view_only=f61a8e8742394561b6e18f40e5051f51.
